# From novice to expert: preparing your peer review

**DOI:** 10.1128/mbio.00430-25

**Published:** 2025-06-05

**Authors:** Diana M. Proctor, Rachy Abraham, Shannon Esher Righi

**Affiliations:** 1 Department of Microbiology and Molecular Genetics, The University of Texas Health Science Center at Houston, McGovern Medical School 12340 , Houston, Texas, USA; 2 Department of Biochemistry and Molecular Biology, Bloomberg School of Public Health, Johns Hopkins University 171261 , Baltimore, Maryland, USA; 3 Department of Microbiology and Immunology, Tulane University School of Medicine 12255 , New Orleans, Louisiana, USA; Johns Hopkins Bloomberg School of Public Health, Baltimore, Maryland, USA

**Keywords:** peer review, training, education, rigor, scientific premise, reproducibility, transparency

## Abstract

Peer review is the process by which the quality of scholarly work is assessed prior to being published, presented, or funded. The consequences of flawed research entering the public domain in the “post-truth” era highlight the need to improve peer review quality, which we believe can be achieved by standardizing training. Here, we aim to enhance the quality of published literature by presenting a systematic guide to train new reviewers (and aid experienced ones) in the art of peer reviewing the rigor of scientific manuscripts.

## EDITORIAL

Peer review is the process that academic journals, conferences, and funding agencies use to ensure the quality of scholarly work before it is published, presented, or funded. Reviewers for academic journals are typically asked to assess whether the investigators rigorously addressed the scientific questions posed, whether the study design is sound, and whether the discussion over-reaches the data. An additional objective of peer review includes determining whether a scholarly product aligns with the journal or funder’s scope, which requires assessment of significance and impact, though the importance of and metrics for determining impact are the subject of debate (1).

In a perfect world, peer review would prevent problematic papers that fail to meet minimal standards of scientific rigor from entering the scientific literature. Yet, peer review has come under increasing scrutiny due in part to a series of high-profile cases of fraudulent or flawed research gaining national media attention (2, 3). Moreover, the growing accessibility of the scientific literature to the public has enabled “influencers” to spread erroneous studies to an audience lacking the context or expertise to critically assess the information. For example, a study published by *The Lancet* in 1998—which was ultimately retracted in 2010—falsely linking the MMR vaccine to autism historically motivated anti-vaccination propaganda and continues to propagate on social media today (4). As early career scientists who trained during the COVID pandemic, we have seen firsthand how low-quality research that enters the public domain pollutes the scientific literature, misguiding public beliefs and policy, and leading to adverse impacts on public health. A recent example is the prominent 2020 study—which was retracted in late 2024—that promoted the use of hydroxychloroquine as a COVID-19 treatment, which led to delays in the development of legitimate therapeutics, as well as adverse effects among patients who took the drug unnecessarily (5). While these problematic papers were ultimately retracted, recent work has highlighted the limited effect that retraction has on changing public opinion (6). As a result, the misuse of flawed, peer-reviewed papers continues to erode public trust in the scientific process, highlighting an urgent need to fortify the evaluation of scientific rigor during the peer review process.

### Introducing a toolkit to train peer reviewers

The consequences of flawed research entering the public domain in the “post-truth” era (7) spotlight the need for increasing peer review quality, which we believe can be accomplished by improved training (8), particularly in evaluating the rigor of a manuscript. Here, we present a systematic guide to train and/or aid scientists in the peer review of manuscripts.

**Fig 1:**
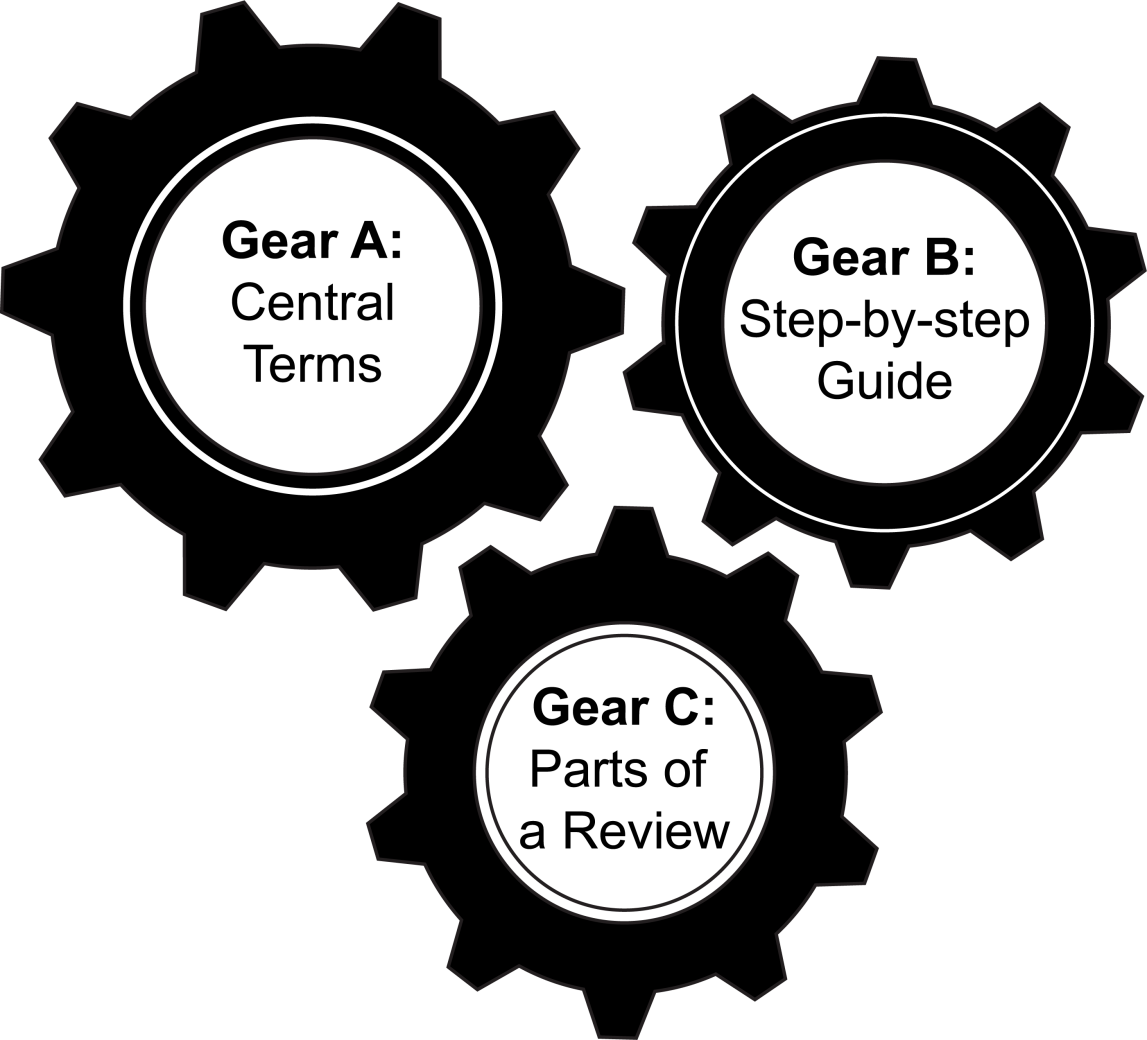
The “gears” driving the process of peer review.

We conceptualize peer review as a process comprised of several critical parts, denoted as gears, since each part is required for peer review to be effective (Fig. 1). For **Gear A,** we start with a set of definitions so that everyone understands what is meant when we use terms such as scientific rigor and significance; a unified understanding is especially important as many of these key terms are the subject of debate (9, 10). **Gear B** is a step-by-step guide with standardized criteria for evaluating manuscripts, as well as a proposed order of operations for first-time reviewers. **Gear C** describes the sections of the review that must be written, as well as the purpose of each section. We believe that setting such community standards will enhance the reliability and quality of the peer review process, making peer review more likely to catch flawed or fraudulent papers before they enter the scientific literature.

### Gear A: defining the terms central to peer reviewing a manuscript

One barrier to improving peer review training is a variable understanding of key terms like rigor, significance, replication, and reproducibility. Indeed, in writing this editorial, we spent considerable time working to clarify our individual interpretations of “reproducible” and “replicable” since we initially had reciprocal definitions of these terms. As a result, existing peer review checklists that do not define these terms may be interpreted differently by different readers, failing to help reviewers approach the evaluation of manuscripts in a systematic manner. To address this, we include as our first gear of peer review a table of key terms along with guidance on how to assess them (Table 1). For readers interested in great debates surrounding the usage of these terms, we also include references, some of which define these terms differently than we do. By defining our usage of these key terms up front, we aim to reduce ambiguity in our guide, giving reviewers an actionable framework for assessing manuscripts systematically.

**TABLE 1:** Definitions of terms central to the process of peer reviewing a manuscript and suggestions for their evaluation

Term	Definition and how to evaluate it
Rigor	According to the NIH (11), rigor is the strict application of the scientific method to ensure robust and unbiased experimental design, methods, analysis, interpretation, and reporting. This requires identification of biological variables, such as sex, and how they are factored into research designs/analyses. Reviewers should evaluate the following key aspects of rigor (12–15): 1. Clear hypothesis or question For hypothesis-driven studies that seek to identify a causal relationship, check that the hypothesis presents a testable relationship between the independent (predictor) and dependent (outcome) variables. For descriptive studies that aim to characterize or summarize phenomena, check that the study clearly defines the variables being observed and provides a clear framework for data analysis. Ensure that the study does not imply a causal relationship where it has not been demonstrated. Check that the authors take steps to mitigate bias (16). Strategies to minimize bias include randomization, blinding, standardized, or pre-defined protocols for data collection, analysis, etc. Check that the authors acknowledge the limitations of their investigation. Limitations may be relevant to study design, study population, or methodology, including identifying factors that likely affect generalizability. 2. Inclusion of controls (17) Check for the inclusion of appropriate negative and positive controls. Negative controls quantify background noise and are essential to prevent noise from being erroneously attributed to biology. Even DNA sequencing experiments benefit from negative controls, which ensure samples are not contaminated. Positive controls ensure the assay was set up correctly and can serve many purposes, including validating experimental conditions, comparing to a test group, or standardizing data across experiments. 3. Inclusion of replicates Check for the inclusion of biological and/or technical replicates, as applicable. Biological replicates represent parallel measurements of biologically distinct samples that help estimate biological variation. For example, in a qPCR or ELISA, one might include biological replicates taken from three to five distinct mice subjected to the same condition to query the effect of a treatment. Technical replicates are repeated measurements of the same sample and help control for variation in the measurement/technical process. In a qPCR or ELISA, a single biological sample will be repeated two to three times using the same experimental settings. Consider steps for which replication is necessary. You can replicate any step in a protocol, not just the initial collection or final sequencing, for example. 4. Appropriate statistical support If models are used, check whether authors state their assumptions clearly and test the sensitivity of their conclusions to departures from assumptions. Where appropriate, check that the authors demonstrate the data meet the statistical assumptions of the tests used. Check if the sample size is sufficient to identify an effect, particularly when null findings are reported (typically, achieved power should be reported in this case). Check to ensure authors avoid pseudoreplication (18) where replicates are treated as independent data points. Common sources of pseudoreplication include time points or spatial series. For example, in a microbiome survey, N is typically the number of human hosts, not the sum of the number of biological samples derived from each human. Check that the authors do not conflate correlation with causation. Check for the interpretation of p-values. Check that the authors report effect sizes (e.g., F-statistics, Pearson’s correlation coefficients, odds ratios, log-fold changes, etc.) as well as p-values, so “significance” can be interpreted. Check whether authors correct for multiple testing, where multiple statistical tests are performed on the same data. Check that the authors specify the statistical test when reporting effect sizes and p-values. Check your strengths and limitations as a reviewer. Write a note to the editor recommending statistical review if you suspect statistical flaws but cannot explain how the errors impact rigor (19).
	5. Experimental replication (20) Check for redundancy in experimental design (14) to assess the likelihood that findings are generalizable. Examples include: Use of multiple methods that are robust to statistical assumptions. Use of multiple cell lines or strains. Use of independent data sets, including published ones. Check to see if the authors perform direct replication (21) within the study. In other words, did they conduct the entire experiment more than once (often denoted as the number of independent experiments)? Note this is distinct from including biological or technical replicates. Refinement experiments (10), in contrast to direct replication experiments, modify or control for a variable identified as important in an initial study. Refinement can serve a role similar to direct replication within a single report, increasing confidence that the study can be replicated by others. This is sometimes referred to as reproducibility in usage (22).
Transparency and reproducibility	According to the NIH, transparency (11) refers to the openness and clarity with which information is shared, particularly regarding research processes and results. Consideration of sex and other biological variables is an inherent component of transparency, as is a clear articulation of details of experimental and computational protocols required for independent investigators to redo the study. Research that fails transparency tests cannot be reproduced—where we define reproducibility as the ability of a different analyst to arrive at the same results given the same data and code—or replicated, making transparency critical. Peer reviewers should assess the following when evaluating transparency: 1. Transparent data reporting (23) Check the axes are clearly labeled and that the scale does not exaggerate effects. Check that authors report on how missing data or outliers are handled. Check that the type of error bar is disclosed (24). Check for raw data distributions and/or reporting Are raw data distributions shown alongside summary statistics in figures (e.g., bar plots and boxplots) so the reader can assess variation in measurements and for effects like bimodality/outliers? Check that the authors state whether the data presented in the figure are representative of or cumulative across experiments. 2. Transparency of methods Check to see if the study could be replicated either directly or conceptually using the experimental methods presented. Check to ensure that key biological or chemical resources that may differ from lab to lab (e.g., cell lines, specialty chemicals, antibodies, and other biologics, not standard laboratory reagents) are fully defined. Best practices (25) suggest that measures demonstrating the authenticity of the resource should be included. 3. Code availability (26) Check to make sure the authors provide access to the code used to generate the results. If this is not provided, make sure that tool parameters and arguments are fully detailed in the methods. Check to make sure methods report software/hardware dependencies, as well as version numbers of bioinformatics tools, software packages, and/or databases. Reviewers should ask the authors to report hard-coded values or parameters in code that could lead to inconsistencies when re-run by others, including instances where randomization is invoked. 4. Data availability For data where there are data sharing mandates, ensure the raw data are uploaded to public repositories. For all experiments, check that details on data preprocessing, cleaning, and/or filtering used to turn raw data into the source data underlying figure displays have been included.
Scientific premise	The scientific premise is the framing of the research question. The peer reviewer should evaluate the strengths and weaknesses (or rigor) of the prior cited work, which the authors used to support their argument for undertaking the work presented in their manuscript. Peer reviewers should evaluate the introduction and Discussion as follows: Is the present study the most logical next step, based on the presentation of the literature? Are there any gaps in logic the authors must articulate to support their argument? Are seminal or contradictory findings in the literature adequately presented and resolved? Are the studies the authors cite rigorous (see definition of rigor) and representative of the field? Are the authors presenting a biased view of the literature surrounding the scientific question? Are citations correct? Are there excessive self-citations?
Significance	Authors typically present the significance of their work in the introduction where their scientific premise is defined, as well as in the Discussion, where they contextualize their work in the field. A significant (27) finding is one that changes the way that we view fundamental processes in fields such as biology, physics, or chemistry. To evaluate significance, you might ask, why does it matter? Does the report improve our understanding of a disease or a fundamental process in biology, physics, or chemistry? Refer to the journal scope for its significance requirement and weigh the significance section in your review accordingly.
Impact	Impact is often understood as the ability of a finding to change the way we do something—how the work can be applied in the field, or where there are broad impacts in related fields. A work can be impactful, but not significant. For example, publishing a 16S rRNA gene database is highly impactful but may or may not be significant. On the other hand, elucidation of the gene required to encode green fluorescent protein (GFP) (28) is both significant (elucidating basic biology) and impactful (can be applied to other fields). To evaluate impact, you might ask, “So what?” Does the research have the potential to lead to new treatments, policies, practices, or applications? How likely is the report to be translated to a broader audience? Refer to the journal scope for its impact requirement and weigh the impact accordingly.

### Gear B: step-by-step guide to evaluating a manuscript

**Fig 2:**
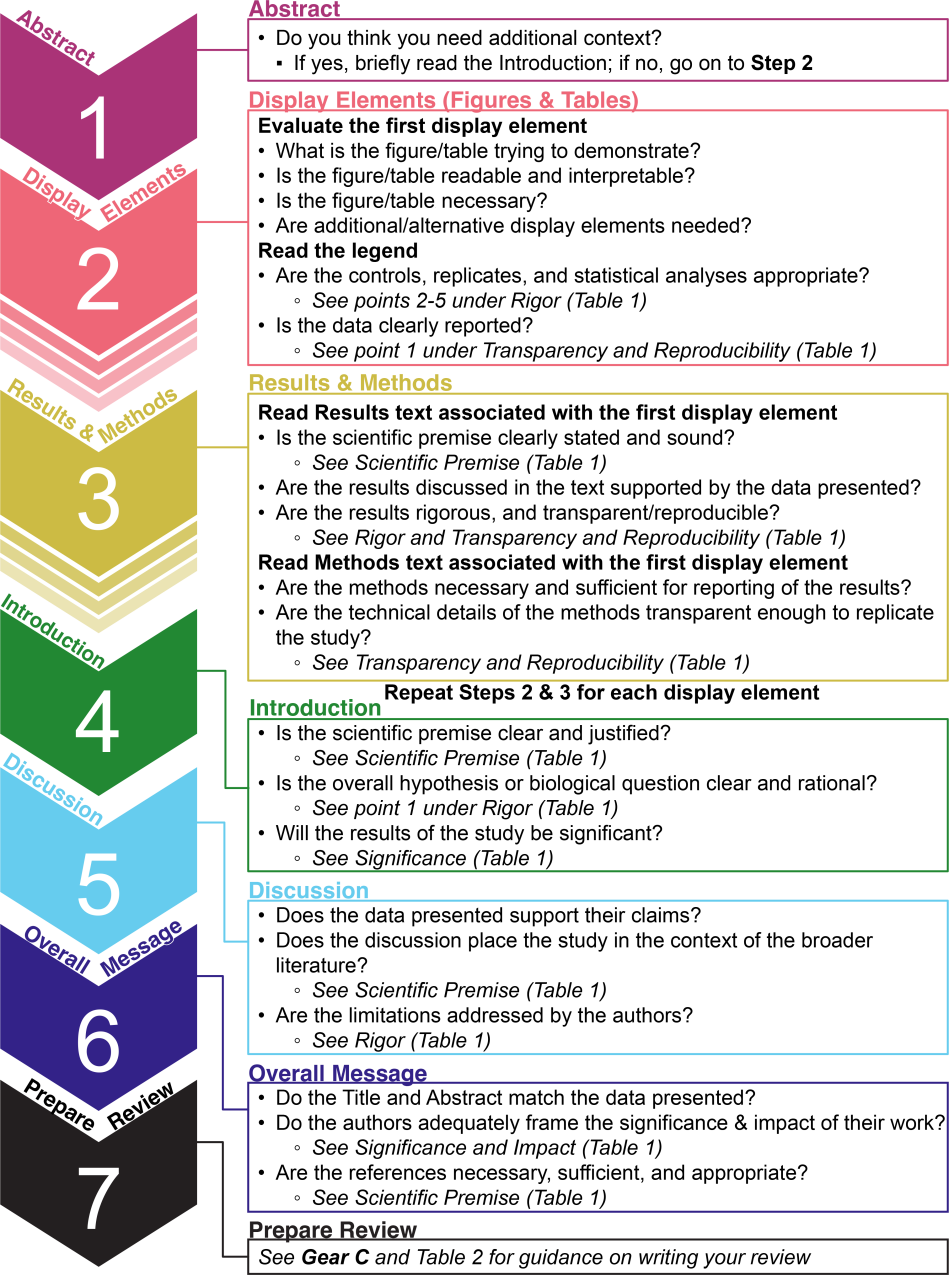
Step-by-step guide for evaluating a manuscript.

An additional issue is that scientists learn to peer review informally, through mentorship and on-the-job experience, without standardized training, resulting in reviews with widely varying quality. Moreover, many reviewers focus their critiques on the English style or grammar of the manuscript—tasks typically managed by copy editors—rather than on its scientific rigor. This can be discouraging and counterproductive to the training of scholars, particularly those for whom English is not a first language (29). Consequently, to help investigators navigate the process of evaluating a manuscript, we have developed a step-by-step guide (Fig. 2) that outlines what to read, assess, and write, and in what order. The step-by-step guide includes a series of questions, framed around the terms presented in Table 1 , to aid in manuscript evaluation. By linking this guide to the definitions provided in Table 1 , we aim to bring greater clarity to reviewers on how to evaluate the scientific rigor of a manuscript, as well as greater precision when training new reviewers.

### Putting the gears in motion: the mechanics of reviewing

**Step 1** in our guide is to read (or re-read) the abstract. If upon reading the abstract you feel that you need more context before proceeding, we advise that you briefly read the introduction. **Step 2** is to evaluate the first display element (e.g., figure or table) in the manuscript. Begin with the figure/table itself and assess its message, interpretability, and necessity before moving on to the legend. Evaluate whether the data have been generated and presented in a rigorous fashion. It can be helpful to review a guide on what to inspect in an image or figure/table (30, 31). **Step 3** begins with reading the Results narrative associated with the display element in question and assessing its scientific premise, rigor, and transparency, as well as evaluating the extent to which the data presented support the authors’ interpretations or claims. Along with the Results text, we also advise that the Materials and Methods text associated with the display element be evaluated in this step; when reading the Materials and Methods, consider whether they are transparent enough for an expert to redo the study. Review supplementary display elements as they are referenced in the text. Steps 2 and 3 should then be repeated for each display element presented in the main manuscript. **Step 4** is to read the introduction in detail, evaluating the scientific premise, significance, and references. **Step 5** is to read the Discussion in detail, evaluating the extent to which the data presented support their claims, and whether the authors have considered the broader context and limitations of their study. **Step 6** is to consider the main message of the manuscript. Are the conclusions supported by the data reflected in the abstract and in the title of the manuscript? Do the title and abstract reflect the limitations of the work? For example, if a study was done in mice, does the title reflect this? Do the authors adequately frame the significance and impact of their work, or alternatively, do they overgeneralize their findings? Along those lines, evaluate whether the references cited by the authors are appropriate, sufficient, and representative of the relevant literature in the field. **Step 7** is to prepare your review, which will be discussed in more detail in the following section.

### Gear C: understanding the “parts” of a peer review report

Preparing the peer review report to submit to the journal is perhaps the most undefined aspect of the peer review process. Although some journals now provide feedback on how to format your peer review report, many simply provide an open-ended text box for the reviewer. Despite this, most reviewers divide their reports into discrete sections or “parts,” i.e., an introductory paragraph, major points, and minor points. As members of the *mBio* Early Career Editorial Board (ECB) (32), we have heard from multiple ASM journal editors that reviewers often confuse major and minor points. Furthermore, many new reviewers are unsure of what to include in the confidential note to editors, and this section is therefore often under-utilized. To clarify these points, this section will walk through the purpose of the common “parts” of a peer review report and provide examples of what should and should not be included in each section (Table 2).

**TABLE 2:** “Parts” of a peer review report^*a*^

Review part	Purpose	What to include	What not to include
Introductory paragraph	Conveys to the authors that you understood the manuscript; conveys to the editor the perceived significance and impact of the work	Summarize Key findings Major advances Major weaknesses	Recommendation to accept or reject^*b*^
Major points^*c*^	Communicates fatal flaws that must be addressed before the paper can be accepted for publication	Changes that are essential to support the claims made in the manuscript, such as: Experiments Controls Analyses Reinterpretations	Additional work that will extend the manuscript beyond its current scope
Moderate points^*d*^	Communicates non-experimental points that will strengthen the manuscript and which may be required to establish rigor	Non-experimental additions or changes that will strengthen the manuscript, such as: Comments on the transparency and reproducibility of the methods Tone, pacing, and contents of the introduction or Discussion	Comments that are considered minor points, which do not greatly impact the overall decision (see below)
Minor points	Communicates minor errors or technical issues that do not greatly impact the overall decision	Minor issues, such as: Imprecise or confusing words Minor mistakes or typos Missing, incorrect, or inappropriate citations Errors that can be referenced easily by line numbers	Comments on the language or grammar^*e*^ Stylistic concerns
Confidential notes to the editor	Candid assessment of the manuscript’s strengths and weaknesses, your qualifications as a reviewer, and suggested decision/action to be taken	Information you wish to communicate to the editor, such as: Rationale for the decision, including if there are any fatal flaws in the manuscript Qualifications (or lack thereof) to review the manuscript, or aspects of the manuscript, in question Qualifications (or lack thereof) to assess the appropriateness of citations	Comments intended for the author Concerns about scientific misconduct^*f*^

^*a*^ Adapted from references 33, 34.

^*b*^ While many journals request that you do not directly state your decision in the comments to the authors, check the specific requirements of each journal.

^*c*^ Some journals provide specific recommendations regarding how the number of major points or requested experiments relates to your final decision.

^*d*^ Some reviewers choose to combine major and moderate points.

^*e*^ If the language or grammar prohibits you from being able to interpret the data, it is recommended that you contact the editor or journal staff prior to conducting your review.

^*f*^ Concerns of this nature should be communicated directly to the journal staff or editor ahead of review submission.

### Putting pen to paper: writing the peer review report

Regardless of the article’s overall quality, begin your peer review report by writing an **introductory paragraph**, which should summarize the key findings, major advances, and major weaknesses of the manuscript. The purpose of the introductory paragraph is to convey to the authors and the editor(s) what you understood from the manuscript. Acknowledging the work the authors invested in the paper, focus on maintaining a civil and collegial tone. Also consider mentioning in the introductory paragraph whether the article represents a valuable addition to the literature and if its findings have wide-reaching implications for future research in related areas.

Next, we suggest outlining **major points**, which are issues with the rigor of the manuscript that must be addressed before the manuscript should be accepted for publication. These may include additional experiments, controls, or analyses that are essential to support the claims made in the manuscript. Alternatively, you may suggest that the authors reinterpret or take a step back on their claims to support the data presented in the manuscript. It is important to consider what must be done versus what you as the reviewer would have done or suggested had you been a co-author. Avoid requesting additional work that will extend the manuscript beyond its current scope.

Third, we suggest writing **moderate points**, which communicate concerns that will strengthen the manuscript, but are not directly relevant to scientific rigor. These can include comments on the reporting of the methods (which may indirectly relate to rigor), as well as the tone, pacing, and content of the introduction and Discussion. Finally, we suggest writing **minor points**, which are errors or other issues that do not greatly impact the overall decision but still warrant attention. It is important to ensure that references are accurately cited and, if not, noted in this section of your review. This includes verifying that the cited work directly supports the claims made and that there is no inappropriate self-promotion through citation. A thorough review of citations helps prevent bias and misrepresentation of sources in the manuscript. Importantly, comments related to language and grammar are not appropriate, nor are purely stylistic concerns.

Following the comments to the author, there is an opportunity to submit **confidential notes to the editor**. Reviewers are strongly encouraged to utilize this to communicate their candid assessment of the manuscript, including the rationale for their recommended decision as to whether the manuscript should be accepted, revised, rejected, or otherwise. Other information you may wish to communicate are your qualifications, or lack thereof, to assess any specific aspects of the manuscript. For example, if you think there is a problem with the statistical analysis or references, but you are not qualified to determine exactly what, say so.

While some reviewers choose to write their major/moderate/minor points in sentence form, it is completely acceptable to include a bulleted list of points. Subheadings for points related to each figure can also be helpful, as well as references to line or page numbers, as appropriate. Regardless of how you format your review, it is important to clearly differentiate critical points that the authors must address from minor concerns/errors.

Prior to submitting your final review, we recommend that you read through it a final time to ensure brevity, collegiality, and civility. Additionally, ensure that you do not make any imprecise demands, which can be identified by looking for an over-abundance of adjectives in your review. For example, instead of simply saying a study is “too descriptive” (35), state what specific experiments or analyses the authors would need to conduct for the paper to meet the hypothesis-driven standards (36) you have in your mind. Furthermore, it is important to double-check the journal scope to ensure your review aligns with editorial standards and does not include demands that may fall outside those guidelines.

### Other important considerations

It is important to note that our step-by-step guide begins once a review has already been accepted. It is expected that a manuscript review will take upwards of 2–8 h; however, this is generally not done in one sitting. A feature of our step-by-step guide is that each step can be done independently, as time allows. Considerations of whether you should accept a manuscript to review, such as conflicts of interest and time availability, are beyond the scope of this editorial but have been covered in detail elsewhere (33, 37). Additionally, some items that may be considered within the scope of a reviewer, such as checking for research compliance approvals (i.e., IRB, IACUC, IBC, etc.), have been deliberately left out, as many journals now ask specific yes/no questions regarding these approvals. While comments on language or grammar are not appropriate, if you feel that you cannot adequately interpret the data due to an issue with the writing, it is recommended that you contact journal staff and/or the editor to discuss your concerns. Other specific questions that may arise, such as concerns about scientific misconduct, should also be communicated directly to the journal staff and/or editor.

### How do we use these tools in training?

By presenting definitions in conjunction with a step-by-step guide, we aim to provide a toolkit for training/aiding scientists in peer review. Our overarching goal in presenting this toolkit is the creation of a standardized peer review workflow that promotes constructive evaluation of research rigor and transparency. We envision that this toolkit could be used to train undergraduate or graduate students to read papers or to help editorial boards train early-career editors. Critically, we view this toolkit as one that can and should be expanded. As early career scientists, we acknowledge that we have entered several long-standing debates: we do not pretend to resolve them. Rather, we shed light on the concern that many of us interpret commonly used scientific words, such as rigor and replication, differently. Our hope is that the community will build on this resource. Additionally, we designed this toolkit based on papers currently being published, recognizing it will need to evolve as the technologies we use to do science change.

Our workflow has limitations. First, we posit that improving peer review training will improve peer review quality and thereby the rigor of published scientific literature. However, our guide cannot overcome deficiencies in graduate education, nor can it train biologists on all aspects of experimental design and data analysis. To address this, we aim to help reviewers recognize their own strengths and weaknesses and communicate these to the editor through the confidential note. While some reviewers may wish to increase their statistical competency by reading relevant textbooks, taking courses such as Strategies and Techniques for Analyzing Microbial Population Structure (STAMPS) at the Marine Biological Laboratory, and/or collaborating with statisticians in their research, it may be more effective for additional journals to hire statistical editors or deploy AI tools to evaluate the statistical rigor of a manuscript before it is sent out for review. Similarly, journals may wish to use grammar editing tools or engage copy editors to address English grammar and style earlier in the process so that reviewers are more easily able to focus solely on the scientific content. Along those same lines, AI tools could be used to screen display elements for data integrity and fraud (38); to assess the inclusion of critical elements, such as ethics declarations; and to evaluate aspects of transparency and reproducibility, including transparency of methods, data availability, and data reporting. A second limitation is that our toolkit cannot create or modify the scientific culture around peer reviewing. For example, our toolkit will not influence those who view peer review as a gatekeeping function or believe other incentives for reviewing should exist. A third limitation we acknowledge is that many how-to guides and checklists for peer review already exist, but they have not been widely adopted. In our view, this is likely due to their imprecise language, lack of an accompanying step-by-step guide, and lack of infrastructure for training and implementation; however, if widespread adoption is hindered by other factors, simply adding definitions and a system for implementation (e.g., training programs such as the *mBio* ECB [32]) will not resolve this issue, and our toolkit will suffer the same fate. Finally, we wrote this guide to help researchers review primary research articles and recognize that additional guidance on reviewing other manuscript types, such as review articles, will need to be developed in the future.

### Conclusion

As scientists, we pride ourselves on being precise. But, when it comes to reviewing each other’s work, we are very imprecise in our demands and language. The variation in the quality of peer review reports may arise due to the varying perspectives different reviewers bring (in part from their training) to the review process. Each reviewer enforces their individualized standard of what should be included, what is considered appropriate, and what meets the required level of rigor or significance. We aim to bring some precision to the language and methods we use to train peer reviewers, which we hope will increase the rigor of the published scientific literature, safeguarding public trust in the scientific enterprise.
